# Expression of miRNAs in Serum Exosomes versus Hippocampus in Methamphetamine-Induced Rats and Intervention of Rhynchophylline

**DOI:** 10.1155/2018/8025062

**Published:** 2018-02-13

**Authors:** Han-cheng Li, Ying-bo Lin, Chan Li, Chao-hua Luo, Yu-ting Zhou, Jing-ying Ou, Jing Li, Zhi-xian Mo

**Affiliations:** School of Traditional Chinese Medicine, Southern Medical University, Guangzhou 510515, China

## Abstract

**Objective:**

To compare the expressions of miRNAs (microRNAs) in serum exosomes and in hippocampus and to provide insights into the miRNA-mediated relationship between peripheral and central nervous systems in the presence of methamphetamine.

**Methods:**

Published results on conditioned place preference (CPP) in rats conditioned by methamphetamine were replicated. The expressions of miRNAs in serum exosomes and hippocampus were determined by gene-chip sequencing. We then predicted the potential target genes of selected, differentially expressed (DE) miRNAs and then carried out functional analysis of these target genes. We also verified our results by RT-qPCR.

**Results:**

Methamphetamine reward could greatly increase the activity time and distance in the intrinsically nonpreferred side of the behavioral apparatus compared with control rats (*P* < 0.01). Rhynchophylline treatment significantly counteracted these changes (*P* < 0.01). Methamphetamine-induced CPP upregulated 23 miRNAs (log_2_ fold change [FC] > 1, *P* < 0.01) in serum exosomes, whereas rhynchophylline treatment could downregulate these miRNAs (log_2_ FC < −1, *P* < 0.01). Analysis of hippocampal miRNAs profiles found 22 DE miRNAs (log_2_ FC > 1 or <−1, *P* < 0.01). When methamphetamine induced CPP, 11 of those miRNAs were upregulated, whereas rhynchophylline treatment could downregulate these miRNAs. The other 11 miRNAs behaved in the opposite way. We selected six DE miRNAs from each of serum exosomes and hippocampus for target gene prediction and functional analysis. We found that, in both, the DE miRNAs and their target genes may be related to neuronal information transmission and synaptic transmission.

**Conclusions:**

Rhynchophylline blocked the alteration of behavior and the expression of some DE miRNAs induced by methamphetamine. The biological functions of these DE miRNAs target genes are correlated between serum exosomes and hippocampus. As to these biological processes and pathways which are involved in the development of addiction at multiple stages, we speculate that these DE miRNAs in serum exosomes and hippocampus are closely related to methamphetamine addiction.

## 1. Introduction

*Uncaria rhynchophylla* (diào gōu téng; literally: “fish hook vine,” or the cat's claw herb; its Chinese and Japanese name, Gouteng and Chotoko, resp.) is a traditional Chinese medicinal herb. It has been used to lower blood pressure and to relieve various neurological symptoms. Rhynchophylline, a monoamine indole alkaloid, is one of the main effective components of* U. rhynchophylla*. Studies have shown that rhynchophylline can significantly reduce the excitability of the cerebral cortex, with significant antihypertensive, sedative, sleep-inducing, and antispasmodic effects [1–4]. Rhynchophylline has inhibitory effects on the brain L-calcium channel, the central glutamate system, and the cardiovascular system [5–7].

Methamphetamine is an addictive drug that produces long-lasting and severe damage to the central nervous system [8]. At present, the conditioned place preference (CPP) test is one of the most effective methods for the determination of animal reward. Research on drug dependence mainly focuses on rodents and other laboratory animals. A preliminary study found that rhynchophylline can eliminate the CPP effect in methamphetamine-dependent rats, and it is not addictive itself [9].

miRNAs are a class of noncoding, single-stranded RNA molecules encoded by endogenous genes. They play an important regulatory role in the biological development process [10]. Many studies have reported that drug addiction, such as cocaine addiction [11], nicotine addiction [12], opioid addiction [13], and alcohol addiction [14], is associated with miRNAs. Research into the regulatory role played by miRNAs in methamphetamine addiction is in a preliminary stage. Studies have shown that methamphetamine can induce changes in miRNA expression levels, and addiction-related brain miRNAs are directly involved in the regulation of methamphetamine addictive behavior [15]. Therefore, to study the regulation of miRNAs in methamphetamine addiction, it is important to further reveal the addiction mechanism of new drugs and to discover new drug targets.

Studies have shown that some miRNAs are encapsulated in exosomes, which can be picked up by target cells, where they act as the type of endogenous miRNA that silences target genes [16]. As a molecular medium, exosomes can transmit information between cells to cause disease and can carry a variety of substances including protein, mRNA, miRNA, DNA, and lipid. Moreover, exosomes are involved in processes of cell migration, cell communication, angiogenesis, tumor-cell growth, and tumor immunity [17–19]. Another study found that the miRNA expression profiles in serum exosomes were significantly different between ovarian cancer patients and healthy controls. There is a good correlation of miRNA expression between serum exosomes and cancer tissues in ovarian cancer patients [20]. This suggests that disorders of normal activities of miRNAs might cause many diseases.

Therefore, this study replicated a CPP test of methamphetamine dependence in rats to observe the effect of rhynchophylline on methamphetamine-induced CPP. Furthermore, by comparing the differences in miRNA expression between serum exosomes and hippocampus, we explored the miRNA-mediated cross-talk between peripheral and central nervous systems in the presence of methamphetamine.

## 2. Materials and Methods

### 2.1. Animals

Sprague-Dawley rats (weight, 190–210 g; age, 2 months) were supplied by the Experimental Animal Center of Southern Medical University (number SCXK GD 2011-0015, Guangzhou, China). Operating procedures and all animal experimental protocols were in accordance with the guidelines of Southern Medical University and National Institutes of Health (NIH, USA) for laboratory animals' experimental use. The experiments were approved by the Southern Medical University's Experimental Animal Ethics Committee.

### 2.2. Drugs and Reagents

Rhynchophylline (number 1108-20131018, purity 99.8%) was purchased from Matsuura Yakugyo Co., Ltd. (Nagoya, Japan). Methamphetamine (number 1212-9802) was obtained from the National Narcotics Laboratory (Beijing, China). Trizol reagent was purchased from Life Technologies (Carlsbad, California, USA). RNA ScreenTape, RNA Reagent, and High Sensitivity D1000 Screen Tape were purchased from Agilent Technologies Inc. (Paro Alto, California, USA). The Qubit® dsDNA HS Assay Kit (number 4367809) was purchased from Life Technologies (Carlsbad, California, USA). The TruSeq® Small RNA Sample Prep Kit (number 4472908) was purchased from Illumina Corporation (Santiago, California, USA). U6 was used as the calibration gene to provide an internal control. Primers were synthesized by the Guangzhou RuiBo Biotechnology Company (Guangzhou, Guangdong Province, China). All other chemicals that were used in the experiments were of analytic grade.

### 2.3. Apparatus

The CPP instrument was purchased from Shanghai Yu Yan Scientific Instruments Co., Ltd. (Shanghai, China). It is comprised of two equal-sized compartments (30 cm long × 30 cm wide × 30 cm high), one having a white inner part and the other having a black inner part, divided by a wall with a sliding door. Commercial equipment used here was as follows: Agilent 2200 Bioanalyzer, Stratagene Mx3005P Real-time PCR (polymerase chain reaction) instrument (Agilent Technologies, Paro Alto, California, USA); Bio-Rad Gradient PCR (Bio-Rad, Hercules, California, USA); DU800 Nucleic Acid/Protein Analyzer (Beckman, Pasadena, California, USA); IX53 Fluorescence Inverted Microscope (Olympus, Tokyo, Japan); Agilent 2200 TapeStation (Agilent Technologies, Paro Alto, California, USA); ND-1000 Nanodrop (Thermo Fisher, Waltham, Massachusetts, USA); Qubit 2.0 (Life Technologies, Carlsbad, California, USA); and Hiseq 2500 (Illumina company, Santiago, California, USA).

### 2.4. Conditioned Place Preference (CPP) Paradigm

**Figure 1:**
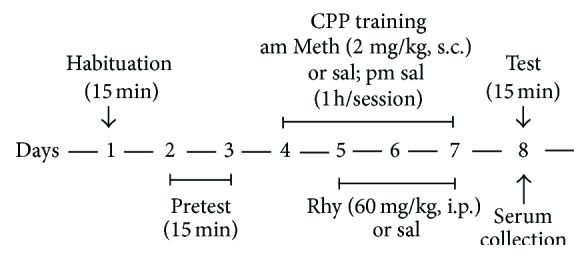
Training process for CPP test.* Notes*. Meth: methamphetamine; Rhy, rhynchophylline; sal: physiological saline.

Rats were tested in a CPP apparatus as described previously. Rats have an innate preference for a black versus a white environment, as confirmed by our pilot test. We therefore identified the white compartment as the nonpreferred compartment (methamphetamine-paired compartment). Rats were tested for baseline preference by walking distance in the nonpreferred compartment during a 15 min trial and calculating the activity time. The rats showing a preference for the white compartment in the preconditioning phase were excluded from further analysis. Suitable rats were divided into three groups of ten randomly, including control group, methamphetamine group, and rhynchophylline treatment groups. The method of the CPP paradigm was copied from our previous reports [21, 22] and is shown in Figure 1.

### 2.5. RNA Extraction and miRNA Microarray Assay

At the end of the CPP test, rats were anesthetized with 10% chloral hydrate, and serum and hippocampi were collected. Serum samples were left standing at 25°C for 2 h and then centrifuged at 1000 ×g for 20 min. Then the total exosome isolation reagent was added, and the tubes were mixed well, placed on ice for 30 min, and centrifuged at 1500 ×g for 10 min at 4°C. All supernatant was decanted to yield the exosome fraction. Exosomes were extracted by adding 1 mL trigene, followed by sonication for 2-3 min at a frequency of 28–35 kHz, which was sufficient for complete dispersal.

After exosome lysis by addition of chloroform, the mixture was shaken and mixed well and let stand for 2 min at RT, followed by centrifugation at 10,000 ×g for 10 min at 4°C. This gave three layers, with RNA in the upper, aqueous phase. The resulting aqueous phase was added to isopropanol, gently and thoroughly mixed, and allowed to stand at −20°C overnight to precipitate RNA, followed by centrifugation at 12000 ×g for 15 min at 4°C to give a pellet of precipitated RNA after complete decantation of the supernatant. The extraction of RNA from the hippocampus was performed in accordance with QIAGEN's miRNeasy Mini Kit manual.

### 2.6. Gene-Chip Sequencing

The Trizol method was used for sample extraction and samples were tested with the Agilent 2200 Tape Station system and the Nano Drop ND-1000 Spectrophotometer. Total RNA (initial volume 1*μ*L) from each acceptable sample was used to construct a miRNA library using the TruSeq® Small RNA Sample Prep Kit (Illumina, San Diego, CA). The main steps of the miRNA library construction protocol were as follows: (1) 3′ adaptor connection; (2) 5′ adaptor connection; (3) first strand cDNA synthesis; (4) PCR amplification; (5) fragment size selection; and (6) quality and quantity evaluation in an Agilent 2200 Tape Station (acceptable: *A*260/*A*280 ≧ 1.8, *A*260/*A*230 ≧ 1.0, and RIN [RNA integrity number] ≧ 7.0). Then the library of acceptable miRNAs was submitted to sample preparation according to the method described in the lllumina HiSeq 2500 User Guide, the final concentration of sample being 10 pM.

### 2.7. Validation of Selected DE miRNAs by RT-qPCR

The total RNA in serum exosomes and hippocampus from each of the three groups was extracted according to the above RNA extraction method. Then 1*μ*L total RNA from each sample was taken and synthesized into cDNA by reverse transcription according to the manual of the reverse transcription kit. One microliter of reverse-transcribed cDNA was taken and evaluated according to the instructions for SYBR® Select Master Mix dye by real-time PCR. The 7900HT Fast Real-time PCR System was used to amplify the different genes. The program used for amplification was as follows: 2 min at 50°C, 2 min at 95°C, followed by 40 cycles of 15 s at 95°C for denaturation, and 1 min at 60°C for annealing and extension. U6 was used as a reference gene. Three independent experiments were performed.

### 2.8. MiRNA Sequence Analysis, Target Gene Prediction, and Functional Analysis

The expression of miRNAs in each library was estimated using the free web-server tool sRNAbench, where the normalized read count of each miRNA was shifted by the following formula: RPM = (miRNAs read number/total map reads) × 1,000,000. Evaluation of differential expression (DE) of miRNAs was employed to focus our sugar-edge study. miRNAs were characterized by having log_2_ FC > 1 or < −1, *P* < 0.01 [23]. Target Scan, DIANA Tools, and miRDB (microRNA database) were used to predict target genes of chosen DE miRNAs. To exclude false-positive predictions, the candidate target genes were the intersection set of all three gene-prediction programs' outputs.

GO (gene ontology) and KEGG (Kyoto Encyclopedia of Genomes and Genes), accessed on the David site (https://david.abcc.ncifcrf.gov), yielded the miRNA targets of hippocampus and serum exosomes, respectively. The GO categories in this study included biological processes, cellular components, and molecular functions. KEGG was used to acquire an overview of regulatory and metabolic pathways. The top 10 GO terms and KEGG pathways were identified. A threshold of *P* < 0.05 was used to enrich each miRNA's significant biological functions.

### 2.9. Statistical Analysis

Data were presented as mean ± standard deviation and analyzed by one-way analysis of variance, followed by the two-tailed, least-significant-difference post hoc test. Statistical analyses were carried out by SPSS software (version 19.0). *P* values < 0.05 were regarded as statistical significance.

## 3. Results

### 3.1. Rhynchophylline Blocked the Behavioral Change Induced by Methamphetamine

**Figure 2:**
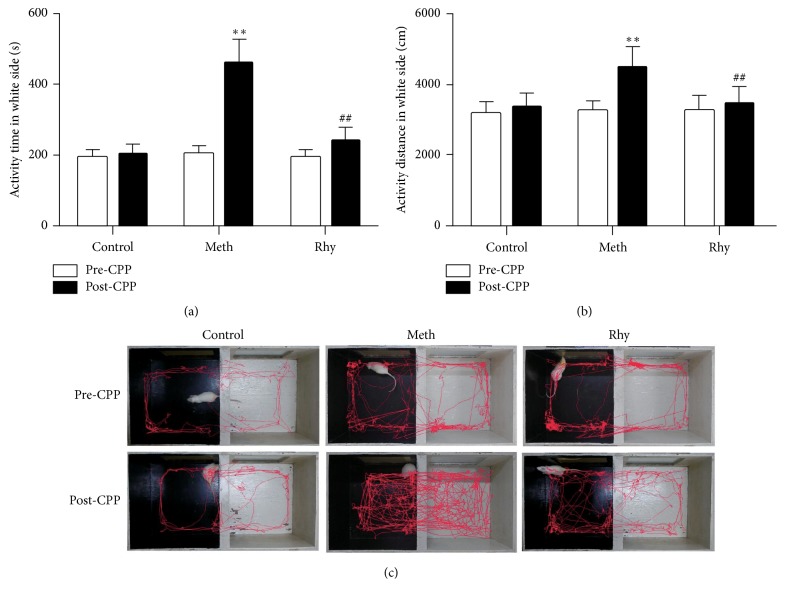
The effect of rhynchophylline on methamphetamine-induced CPP in rats. (a) Change of activity time of rats in nonpreferred compartment induced by methamphetamine. (b) Change of activity distance of rats in nonpreferred compartment induced by methamphetamine. (c) Activity routes of rats in CPP compartment. Control, control group; Meth, methamphetamine group; Rhy, rhynchophylline treatment group; *n* = 10; ^*∗∗*^*P* < 0.01 versus control group; ^##^*P* < 0.01 versus methamphetamine group.

Rhynchophylline's effect on methamphetamine-induced CPP rats is shown in Figure 2. The movement distance and activity time of rats in nonpreferred compartment was greatly increased by methamphetamine compared with that of the control rats (*P* < 0.01), whereas rhynchophylline decreased the movement distance and activity time significantly and providing values like those in the control group (*P* < 0.01). Rat activity routes in CPP are shown in Figure 2(c). In comparison with those of the control rats, the activity routes in the nonpreferred compartment were increased greatly by methamphetamine. But rhynchophylline reduced the activity routes significantly.

### 3.2. Screening of DE miRNAs in Serum Exosomes and Hippocampus

**Figure 3:**
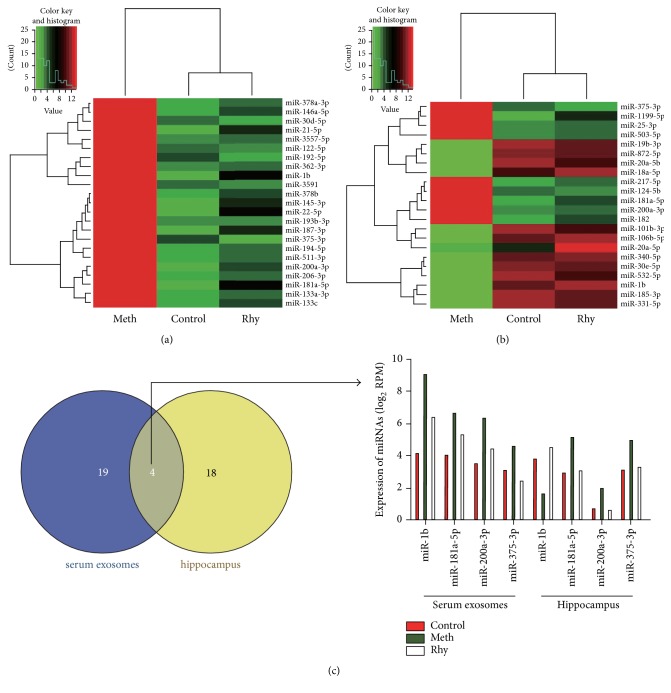
Heat map and Venn diagram of DE miRNAs in serum exosomes and hippocampus of rats after CPP. (a) A heat map showing 23 DE miRNAs in serum exosomes of CPP rats. (b) A heat map showing 22 DE miRNAs in hippocampus of CPP rats. (c) A Venn diagram showing the relationship between DE miRNAs in serum exosomes and hippocampus.

The alterations of miRNA expression in serum exosomes were determined by gene-chip sequencing with the following results. Compared with the control group, methamphetamine upregulated 50 miRNAs (log_2_ FC > 1, *P* < 0.01) and downregulated 2 miRNAs (log_2_ FC < −1, *P* < 0.01). Compared with the methamphetamine group, rhynchophylline downregulated 32 miRNAs (log_2_ FC < −1, *P* < 0.01) and upregulated 9 miRNAs (log_2_ FC > 1, *P* < 0.01). What is more interesting, when methamphetamine induced CPP, 23 of those miRNAs were upregulated, whereas rhynchophylline treatment could downregulate these miRNAs (Table 1, Figure 3(a)).

The alteration of miRNA expressions in the hippocampal area was also determined by gene chip and we found 22 DE miRNAs (log_2_ FC > 1 or < −1, *P* < 0.01) (Table 2, Figure 3(b)). When methamphetamine induced CPP, 11 of those miRNAs were upregulated, whereas rhynchophylline treatment could downregulate these miRNAs. The remaining 11 miRNAs behaved oppositely. Twenty-three DE miRNAs in serum exosomes and 22 DE miRNAs in hippocampus were further analyzed by Venn diagram, and we found that four miRNAs were DE co-miRNAs (Figure 3(c)). However, the expressions of these four miRNAs in the serum exosomes and hippocampus were not exactly the same, suggesting that methamphetamine induction of miRNA DE is tissue-specific. However, rhynchophylline could block the DE of miRNAs induced by methamphetamine whether in serum exosomes or in hippocampus.

### 3.3. Target Gene Prediction for Six DE miRNAs and Their Functional Analysis

**Figure 4:**
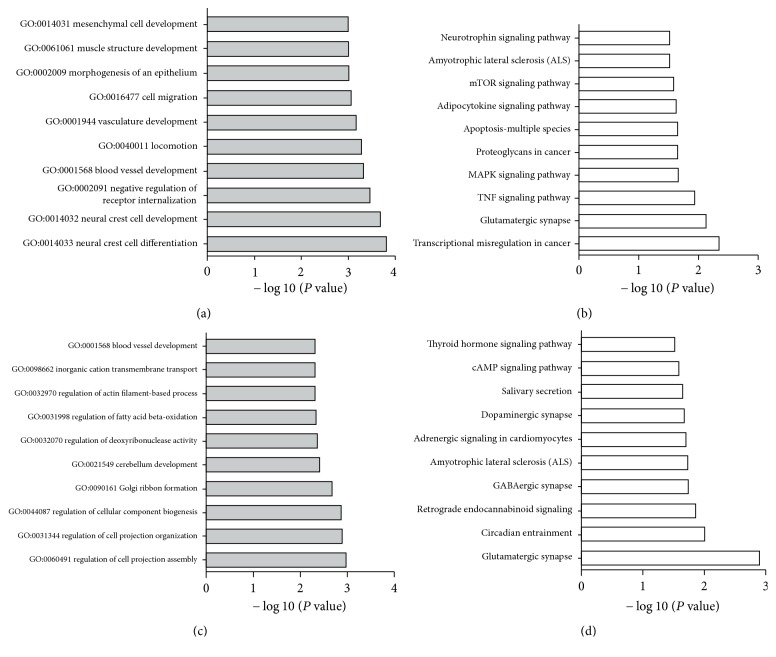
GO and KEGG pathway analyses of predicted targets of selected DE miRNAs. (a) Top 10 sorts enriched by GO for serum exosomes. (b) Top 10 sorts enriched by KEGG pathway analysis for serum exosomes. (c) Top 10 sorts enriched by GO for hippocampus. (d) Top 10 sorts enriched by KEGG pathway analysis for hippocampus.* Notes*. The *y*-axis: the names of GO or KEGG, the *x*-axis: the values were presented by −log_10_ (*P* value); the smaller the *P* value, the larger the value of −log_10_ (*P* value).

Based on the above analysis, we selected six DE miRNAs from each of serum exosomes and hippocampus for target gene prediction. In serum exosomes, a total of 962 targets of rno-miR-145-3p, rno-miR-181a-5p, rno-miR-21-5p, rno-miR-200a-5p, rno-miR- 375-3p, and rno-miR-1b were predicted by Target Scan, miRDB, and DIANA Tools. These candidate targets were divided into 322 kinds according to the biological processes of the GO categories (*P* < 0.05), specially included neural-crest cell differentiation, neural-crest cell development, blood vessel development, and negative regulation of receptor internalization. The top 10 classes enriched by the GO categories are shown in Figure 4(a). For KEGG pathway analysis, the candidate targets' genes were primarily located in transcriptional misregulation in cancer, glutamatergic synapse, TNF signaling pathway, MAPK signaling pathway (MAPK, mitogen-activated protein kinase), and so on, indicating that the DE miRNAs and their target genes are related to cancer, neuronal information transmission, gene expression regulation, cell apoptosis, and synaptic transmission (Figure 4(b)).

In hippocampus, a total of 475 targets of rno-miR-101b-3p, rno-miR-217-5p, rno-miR-375-3p, rno-miR-20a-5p, rno-miR-19b-3p, and rno-miR-182 were predicted. These candidate targets were divided into 258 kinds according to the biological processes of the GO categories (*P* < 0.05), specially included regulation of cell-projection assembly, regulation of cell-projection organization, regulation of cellular-component biogenesis, Golgi ribbon formation, and cerebellum development. The top 10 classes enriched by the GO categories are shown in Figure 4(c). For KEGG pathway analysis, the candidate targets' genes were primarily located in glutamatergic synapse, circadian entrainment, retrograde endocannabinoid signaling, GABAergic synapse (GABA, gamma-aminobutyric acid), and so on, also indicating that the DE miRNAs and their target genes are related to neuronal information transmission, synaptic transmission, and neuroprotection (Figure 4(d)). In comparing the top 10 KEGG pathways between serum exosomes and hippocampus, one copathway was found, such as glutamatergic synapse.

### 3.4. Validation of miRNAs by RT-qPCR

**Figure 5:**
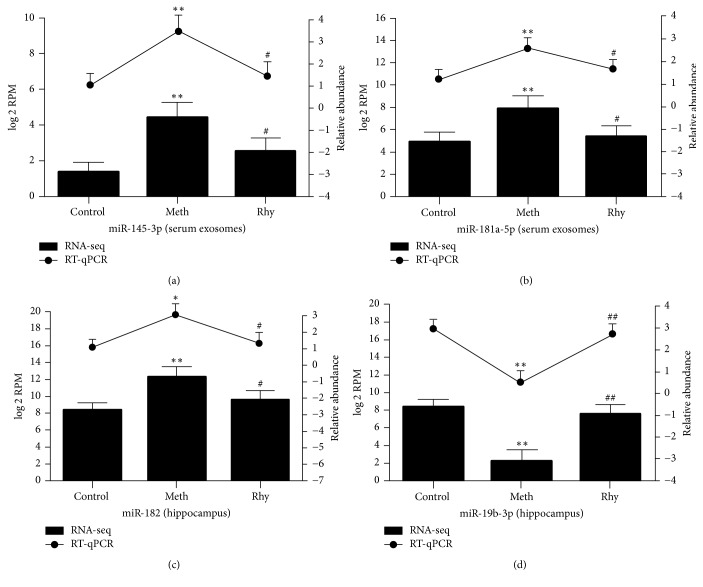
DE of Selected-MiRNAs Detected by RT-qPCR and MiRNA-seq. (a) Expression of miR-145-3p in serum exosomes. (b) Expression of miR-181a-5p in serum exosomes. (c) Expression of miR-182 in hippocampus. (d) Expression of miR-19b-3p in hippocampus. miRNA expression from RT-qPCR (quantitative, reverse-transcription polymerase chain reaction) is represented by lines on the top and values are shown on the right vertical axis as relative abundance. miRNA expression data from miRNA-seq is represented by bars on the bottom and values are shown on the left vertical axis as log_2_ RPM (normalized reads number). ^*∗∗*^*P* < 0.01; ^*∗*^*P* < 0.05 versus control group. ^##^*P* < 0.01; ^#^*P* < 0.05 versus methamphetamine group. Data are presented as mean ± standard deviation.

To further verify the accuracy of our gene-chip analysis, we selected 2 DE miRNAs from each of serum exosomes and hippocampus for verification by RT-qPCR (quantitative, reverse-transcription polymerase chain reaction). The dissolution-curve analysis showed no heterozygotes, indicating that the amplification products were single and free from nonspecific amplification. The amplification curve indicated that all samples had entered the expansion platform period, indicating that the reaction conditions had been set accurately. From Figure 5, we see that the RT-qPCR results are consistent with those of gene-chip sequencing.

## 4. Discussion

The conditioned place preference test is the classic experiment to detect psychological drug dependence. Our results showed that methamphetamine significantly increased the activity time and distance of rats in the nonpreferred compartment after CPP, suggesting that we have replicated the CPP test successfully. Rhynchophylline decreased the activity time and distance in the nonpreferred compartment after CPP, suggesting that rhynchophylline blocks the behavioral alteration induced by methamphetamine. The results of gene-chip analysis showed that rhynchophylline could block the DE of some miRNAs induced by methamphetamine, whether in serum exosomes or in hippocampus, suggesting that these DE miRNAs are associated with rhynchophylline's ability to modify methamphetamine addiction and withdrawal. After further analysis by Venn diagram, we found four DE co-miRNAs. The expression of these four miRNAs in the serum exosomes and hippocampus is correlated.

miR-181a-5p is one of the four DE co-miRNAs we found. Chandrasekar and Dreyer found that the expression of miR-181a in the nucleus accumbens was upregulated after chronic cocaine administration. They also found that miR-181a in cocaine addiction formed a complex regulatory mechanism that impacted neural plasticity and reward circuits [24]. Saba et al. found that when mice were injected intraperitoneally with amphetamines for 5 days, the expression of mi-181a in hippocampus, nucleus accumbens, and midbrain ventricle was significantly upregulated, especially in hippocampus. Moreover, activation of dopamine signals in hippocampal neurons can induce miR-181a expression. Overexpression and inhibition of miR-181a can regulate synaptic function by negatively regulating GluA2 expression [25]. The study also found that chronic amphetamine and cocaine treatment could upregulate miR-181a expression in specific brain areas of mice. The expression of miR-181a in hippocampus, nucleus accumbens, and the midbrain ventral region was upregulated, the most significant change being in hippocampus, while miR-181a in prefrontal cortex showed no significant change. In contrast, cocaine could significantly upregulate miR-181a in the prefrontal cortex of mice without altering its expression in the hippocampus, nucleus accumbens, or midbrain ventral brain regions, suggesting that different addictive drugs induce miRNAs in different brain regions.

KEGG pathway analysis showed one copathway between serum exosomes and hippocampus, such as glutamatergic synapse. Many studies implicate glutamatergic synapses in drug addiction. Vorel et al. found that electrical stimulation of a hippocampal area that contained glutamatergic fibers elicited glutamate-dependent cocaine-seeking behavior that was localized in the ventral tegmental region, indicating a function for glutamatergic neurotransmission in relapse to cocaine abuse [26]. Lee and coworkers showed that repeated, but not intense, low-dose methamphetamine induces memory damage in mice and the potential mechanism includes decrease of binding to the NMDA (N-methyl-D-aspartate) receptor in specific brain areas that are related to memory and learning [27].

By GO analysis, we found that the biological processes of these DE miRNAs target genes are mainly enriched in neural-crest cell differentiation and development, regulation of cell-projection, and so on. By KEGG analysis, we also found that these target genes are mainly located in glutamatergic synapse, retrograde endocannabinoid signaling, GABAergic synaps, apoptosis-multiple species, and so on. As to these biological processes and pathways which are involved in the development of addiction at multiple stages, we speculate that these DE miRNAs in serum exosomes and hippocampus are closely related to methamphetamine addiction. They may regulate the expression of its downstream target genes and cause the change of biological characteristics.

Rhynchophylline is a calcium-channel blocker and noncompetitive NMDA antagonist [1]. In the central nervous system, rhynchophylline could be good for memory damage that is induced by the disorder of cholinergic systems of the brain [6, 28]. Our previous researches showed that rhynchophylline downregulates the expression of NMDA receptor subtype 2B in the cortex and reverses the rewarding effect of methamphetamine in rats [21, 29]. In the present research, it was found that rhynchophylline regulates the expression of miRNAs in serum exosomes and hippocampus, also reversing the methamphetamine rewarding effect in rats. This means that the addiction mechanism of methamphetamine is complex, involving not only regulation of multiple brain reward pathways, but also the mutual interaction between brain-specific and serum-exosomal miRNAs.

Related studies have found that serum-exosome miRNA expression profiles are significantly different between ovarian cancer patients and healthy persons. Moreover, a good correlation of miRNA expression has been found between serum exosomes and cancer tissues in ovarian cancer patients [20]. Disorders of the normal activities of serum-exosome miRNAs might cause many diseases. Increasing evidence shows that exosomes secreted by peripheral or in vitro cultures can be used for the treatment of central nervous system (CNS) disease. The use of MSC- (mesenchymal stem cell-) derived exosomes in animal stroke models can significantly promote the repair of nerves and improve the prognosis [30]. Huan-Cheng et al. injected exosome secreted by N2a cells into the brain of mice expressing amyloid beta precursor protein (APP) and showed that, in APP mice continuously injected with exosome, the brain concentration of A beta decreased significantly, the formation of A beta plaques decreased significantly, and synaptic plasticity increased significantly [31]. Thus, we speculate that, as a molecular medium, exosomes can transfer information between cells and organs in the process of addiction. Serum-exosome miRNAs are likely to participate in regulatory actions on the brain reward system in addiction. By virtue of their ability to regulate important molecules, these miRNAs may provide new avenues for research on the pathological mechanisms of drug dependence.

## 5. Conclusions

The biological functions of these DE miRNAs and its target genes are correlated between serum exosomes and hippocampus. These DE miRNAs in serum exosomes and hippocampus may cause methamphetamine addiction. They may regulate the expression of its downstream target genes and cause the change of biological characteristics. Or exosomes may be specific biological carriers of miRNAs between peripheral and CNS, mediating an ongoing cross-talk. But further studies of these possibilities are needed. We also showed that rhynchophylline is a promising, natural drug candidate against methamphetamine addiction.

## Figures and Tables

**Table 1:** Twenty-three DE miRNAs in serum exosomes of rats after CPP.

miRNA_ID	Control versus Meth		Meth versus Rhy	
	log_2_ (fold change)	*P* value	log_2_ (fold change)	*P* value
rno-miR-122-5p	4.29	4.47*E* − 06	−4.67	5.56*E* − 08
rno-miR-3557-5p	3.81	1.04*E* − 03	−2.64	1.84*E* − 02
rno-miR-3591	5.03	5.34*E* − 08	−5.44	4.16*E* − 10
rno-miR-21-5p	2.85	6.55*E* − 03	−1.66	1.30*E* − 01
rno-miR-378a-3p	3.19	1.43*E* − 03	−2.72	1.84*E* − 02
rno-miR-146a-5p	3.37	6.05*E* − 04	−2.52	6.01*E* − 03
rno-miR-30d-5p	1.52	4.43*E* − 01	−1.78	9.08*E* − 02
rno-miR-1b	4.92	1.20*E* − 07	−2.60	4.31*E* − 03
rno-miR-192-5p	1.91	1.75*E* − 01	−2.42	9.18*E* − 03
rno-miR-362-3p	2.27	5.99*E* − 02	−2.03	4.16*E* − 02
rno-miR-133a-3p	4.79	3.51*E* − 07	−3.84	9.73*E* − 06
rno-miR-133c	5.11	6.56*E* − 08	−3.97	4.93*E* − 06
rno-miR-181a-5p	2.63	1.77*E* − 02	−1.35	3.02*E* − 01
rno-miR-206-3p	3.37	7.36*E* − 04	−2.79	2.00*E* − 03
rno-miR-200a-3p	2.83	7.96*E* − 03	−1.93	5.97*E* − 02
rno-miR-194-5p	4.75	9.42*E* − 07	−3.81	1.50*E* − 05
rno-miR-511-3p	6.29	5.15*E* − 10	−4.86	6.72*E* − 08
rno-miR-375-3p	1.43	4.45*E* − 01	−2.13	3.51*E* − 02
rno-miR-193b-3p	2.52	3.31*E* − 02	−2.62	5.83*E* − 03
rno-miR-145-3p	3.02	6.08*E* − 03	−1.88	7.96*E* − 02
rno-miR-378b	2.77	1.43*E* − 02	−1.95	6.40*E* − 02
rno-miR-22-5p	2.73	1.72*E* − 02	−1.41	2.76*E* − 01
rno-miR-187-3p	3.45	1.57*E* − 03	−1.99	6.28*E* − 02

**Table 2:** Twenty-two DE miRNAs in hippocampus of rats after CPP.

miRNA_ID	Control versus Meth		Meth versus Rhy	
	log_2_ (fold change)	*P* value	log_2_ (fold change)	*P* value
rno-miR-19b-3p	−3.68	4.16*E* − 10	2.99	7.99*E* − 05
rno-miR-872-5p	−3.40	5.56*E* − 08	2.90	8.47*E* − 03
rno-miR-20a-5p	−3.46	6.72*E* − 08	2.74	1.55*E* − 02
rno-miR-101b-3p	−10.56	9.73*E* − 06	12.99	3.84*E* − 06
rno-miR-106b-5p	−2.78	1.50*E* − 05	2.64	9.18*E* − 03
rno-miR-340-5p	−2.55	6.96*E* − 04	2.21	1.12*E* − 03
rno-miR-30e-5p	−2.19	7.47*E* − 04	2.33	1.16*E* − 02
rno-miR-217-5p	−2.10	1.06*E* − 03	1.93	1.38*E* − 02
rno-miR-1b	−2.18	1.77*E* − 03	2.90	1.50*E* − 02
rno-miR-185-3p	−3.94	1.82*E* − 03	4.26	1.52*E* − 02
rno-miR-331-5p	−1.91	2.00*E* − 03	2.24	1.55*E* − 02
rno-miR-375-3p	1.95	2.51*E* − 03	−1.53	1.68*E* − 02
rno-miR-1199-5p	1.01	2.26*E* − 03	−1.20	1.84*E* − 02
rno-miR-25-3p	2.06	2.83*E* − 03	−1.09	1.31*E* − 02
rno-miR-503-5p	2.08	3.47*E* − 03	−1.50	2.15*E* − 02
rno-miR-18a-5p	3.42	3.84*E* − 03	−2.19	2.19*E* − 02
rno-miR-124-5b	1.31	4.31*E* − 03	−1.15	3.48*E* − 02
rno-miR-181a-5p	1.51	5.83*E* − 03	−1.36	3.51*E* − 02
rno-miR-200a-3p	1.57	6.01*E* − 03	−1.79	4.16*E* − 02
rno-miR-20a-5b	2.08	3.47*E* − 03	−1.50	2.15*E* − 02
rno-miR-532-5p	1.31	4.31*E* − 03	−1.15	3.48*E* − 02
rno-miR-182	1.33	8.47*E* − 03	−1.17	4.26*E* − 02

## Abbreviations

APP: Amyloid-beta precursor protein
CNS: Central nervous system
CPP: Conditioned place preference
DE: Differential expression
FC: Fold change
GABA: Gamma-aminobutyric acid
GO: Gene ontology
KEGG: Kyoto Encyclopedia of Genomes and Genes
MAPK: Mitogen-activated protein kinase
miRDB: microRNA database
miRNA: microRNA
MSC: Mesenchymal stem cells
NIH: National Institutes of Health
NMDA: N-Methyl-D-aspartate
PCR: Polymerase chain reaction
RIN: RNA integrity number
RPM: miRNA read number/total map reads
RT-qPCR: Quantitative, reverse-transcription polymerase chain reaction.
